# Understanding Intracellular Biology to Improve mRNA Delivery by Lipid Nanoparticles

**DOI:** 10.1002/smtd.202201695

**Published:** 2023-06-14

**Authors:** Morag Rose Hunter, Lili Cui, Benjamin Thomas Porebski, Sara Pereira, Silvia Sonzini, Uchechukwu Odunze, Preeti Iyer, Ola Engkvist, Rebecca Louise Lloyd, Samantha Peel, Alan Sabirsh, Douglas Ross-Thriepland, Arwyn Tomos Jones, Arpan Shailesh Desai

**Affiliations:** Advanced Drug Delivery Pharmaceutical Sciences R&D, AstraZeneca Cambridge CB21 6GH, UK; Protein and Nucleic Acid Chemistry MRC Laboratory of Molecular Biology Cambridge CB2 0QH, UK; Advanced Drug Delivery Pharmaceutical Sciences R&D, AstraZeneca Cambridge CB21 6GH, UK; Molecular AI Discovery Sciences R&D, Astrazeneca Gothenburg 431 50, Sweden; Functional Genomics Discovery Sciences R&D, AstraZeneca Cambridge CB4 0WG, UK; Advanced Drug Delivery Pharmaceutical Sciences R&D, AstraZeneca Gothenburg 431 50, Sweden; Functional Genomics Discovery Sciences R&D, AstraZeneca Cambridge CB4 0WG, UK; Cardiff School of Pharmacy and Pharmaceutical Sciences Cardiff University Cardiff CF10 3NB, UK; Advanced Drug Delivery Pharmaceutical Sciences R&D, AstraZeneca Cambridge CB21 6GH, UK

**Keywords:** drug delivery, intracellular trafficking, lipid nanoparticles, machine learning, nucleic acid therapeutics

## Abstract

Poor understanding of intracellular delivery and targeting hinders development of nucleic acid-based therapeutics transported by nanoparticles. Utilizing a siRNA-targeting and small molecule profiling approach with advanced imaging and machine learning biological insights is generated into the mechanism of lipid nanoparticle (MC3-LNP) delivery of mRNA. This workflow is termed Advanced Cellular and Endocytic profiling for Intracellular Delivery (ACE-ID). A cell-based imaging assay and perturbation of 178 targets relevant to intracellular trafficking is used to identify corresponding effects on functional mRNA delivery. Targets improving delivery are analyzed by extracting data-rich phenotypic fingerprints from images using advanced image analysis algorithms. Machine learning is used to determine key features correlating with enhanced delivery, identifying fluid-phase endocytosis as a productive cellular entry route. With this new knowledge, MC3-LNP is re-engineered to target macropinocytosis, and this significantly improves mRNA delivery in vitro and in vivo. The ACE-ID approach can be broadly applicable for optimizing nanomedicine-based intracellular delivery systems and has the potential to accelerate the development of delivery systems for nucleic acid-based therapeutics.

## 1 Introduction

Therapeutic modalities such as nucleic acids require safe and effective delivery to specific intracellular locations to have an effect. Nanosized delivery systems have been engineered to address the challenges of protecting therapeutic nucleic acids from degradation, avoiding clearance mechanisms, distribution to the target tissue, cellular entry, and trafficking to the target subcellular location.^[1]^ Lipid nanoparticles (LNP) are amongst the most well-developed delivery systems, exemplified by the approval of Onpattro (patisiran), the first approved LNP-based nanomedicine for delivery of nucleic acids,^[2]^ and the rapid clinical advancement of LNP-based mRNA vaccines for influenza^[3,4]^ and COVID-19.^[5]^

The type of LNP utilized in Onpattro (MC3-based LNP) results in less than 2% of the cargo reaching the correct subcellular location.^[6]^ Newer LNP designs have increased this to 15%, however most of the cargo is still effectively wasted by being trapped in degradative vesicles such as late endosomes and lysosomes.^[7]^ Due to a limited understanding of the complex of underlying biology of intracellular nanoparticle transport, progress has been largely driven by a materials-centric approach involving high-throughput empirical screening for delivery efficiency. The structure–activity relationships identified by this work has resulted in movement toward more rational LNP design. However, generating a greater degree of biological understanding to inform these activities is an opportunity to accelerate development of safe and effective systems.

Herein we describe a target biology-centric approach to the optimization of MC3-LNP for mRNA delivery termed Advanced Cellular and Endocytic profiling for Intracellular Delivery (ACE-ID). ACE-ID is an approach inspired by modern phenotypic drug discovery processes, in which we used established target identification methodologies to understand key molecular targets, mechanisms, and pathways to exploit for improving the potency of delivery systems. This was done by first treating a model cancer cell line (NCI-H358) with 178 siRNA or 233 small molecules against molecular targets we hypothesized to be relevant for intracellular delivery. We used an image-based functional mRNA delivery assay to identify which molecular targets increase MC3-LNP delivery. Then we designed four assays to measure the effect of siRNA and small molecule treatments on nanoparticle uptake and trafficking mechanisms, to identify specific mechanisms to exploit for improving delivery. Advanced image analysis in combination with machine learning was used to correlate the treatments, which increase functional delivery of LNP with specific phenotypic features in the mechanistic assays. From this we identified macropinocytosis as a target mechanism for improving delivery. Using this insight, we re–engineered the MC3-LNP for improved delivery in vitro and in vivo, demonstrating how ACE-ID can be utilized to optimize nanomedicines for intracellular delivery.

## 2 Results

**Figure 1:**
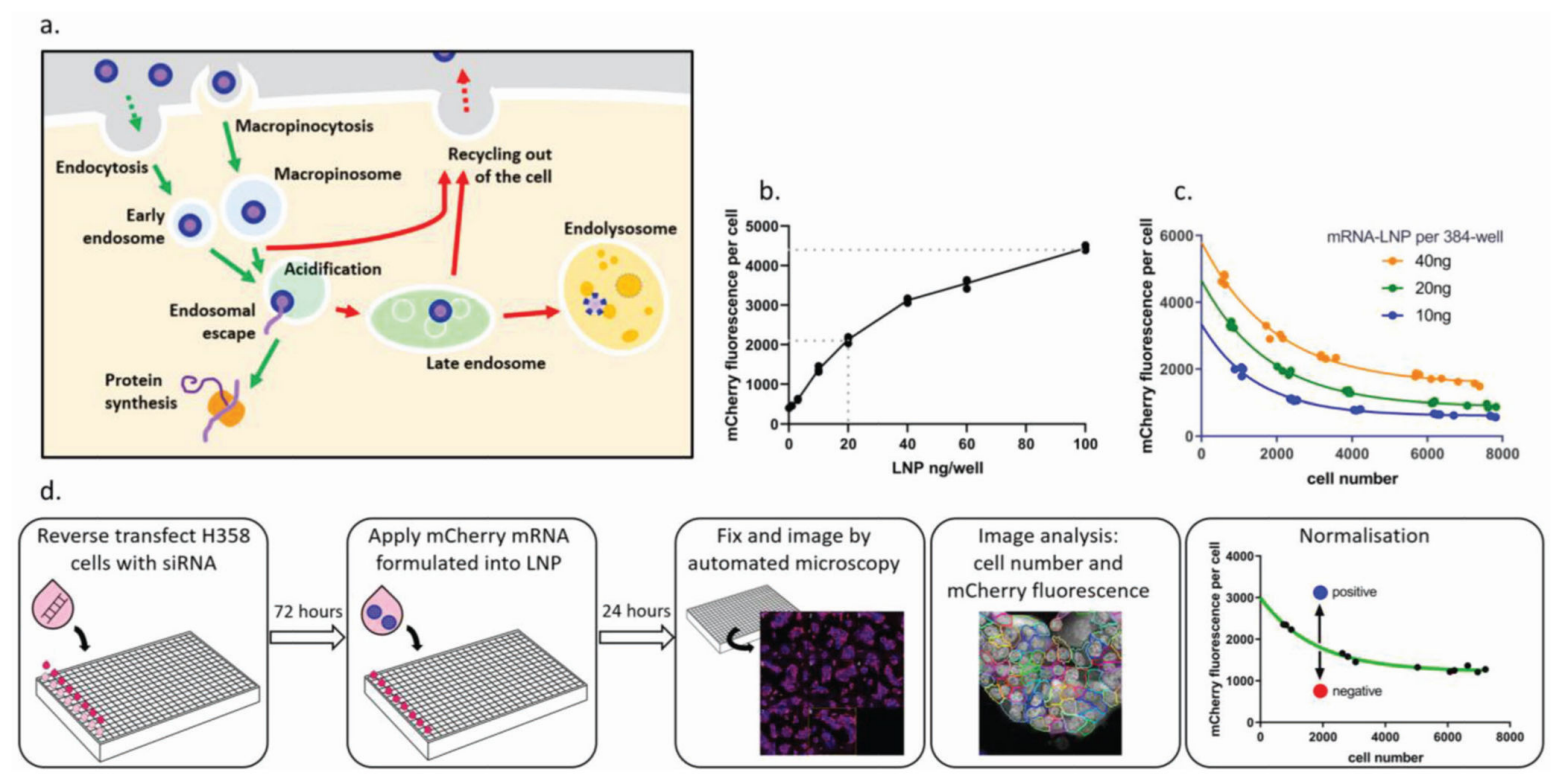
An assay for the functional delivery of mRNA by LNP. a) Functional delivery of mRNA by LNP requires several steps (green arrows), and there are multiple mechanisms by which the delivery process can be disrupted (red arrows). b) Concentration–response of mRNA-LNP in NCI-H358 cells. Black line connects means of three technical replicates per concentration. Gray dashed lines show maximum observed mCherry fluorescence and concentration used in subsequent screening experiments. c) The density of NCI-H358 cells affects the efficiency of mRNA delivery. d) Schematic of the functional delivery screening assay workflow.

The outcome of successful delivery of mRNA using LNP is the production of the protein encoded by the introduced mRNA, termed “functional delivery”. This is the net outcome of multiple distinct processes: LNPs uptake into cells, escape of mRNA from the endosomal system into the cytosol, and the efficiency of mRNA translation to protein (Figure 1a). First, we performed an in vitro screening assay for overall functional delivery, utilizing mRNA encoding the mCherry fluorescent protein, formulated into an MC3-based LNP.^[8]^ Functional delivery of this mRNA results in cytosolic expression of the fluorescent protein, which was imaged by automated confocal microscopy and mCherry expression per cell was quantified. Screens were performed in a cell line, which had previously been identified as having some base-line transfectability with MC3-LNP, enabling us to have a suitable assay window for screening.^[9,10]^ Cells were treated with a moderate concentration of LNP (20 ng per well, 0.4 μg mL^−1^), to allow identification of treatments which increase functional delivery (Figure 1b).

We observed a correlation between cell density and the efficiency of mRNA delivery by LNP, whereby cells at lower densities achieved higher mCherry expression per cell (Figure 1c). To control for effects of siRNA treatments on cell density, mCherry expression per siRNA-treated cell was normalized to cell density (Figure 1d).

**Figure 2:**
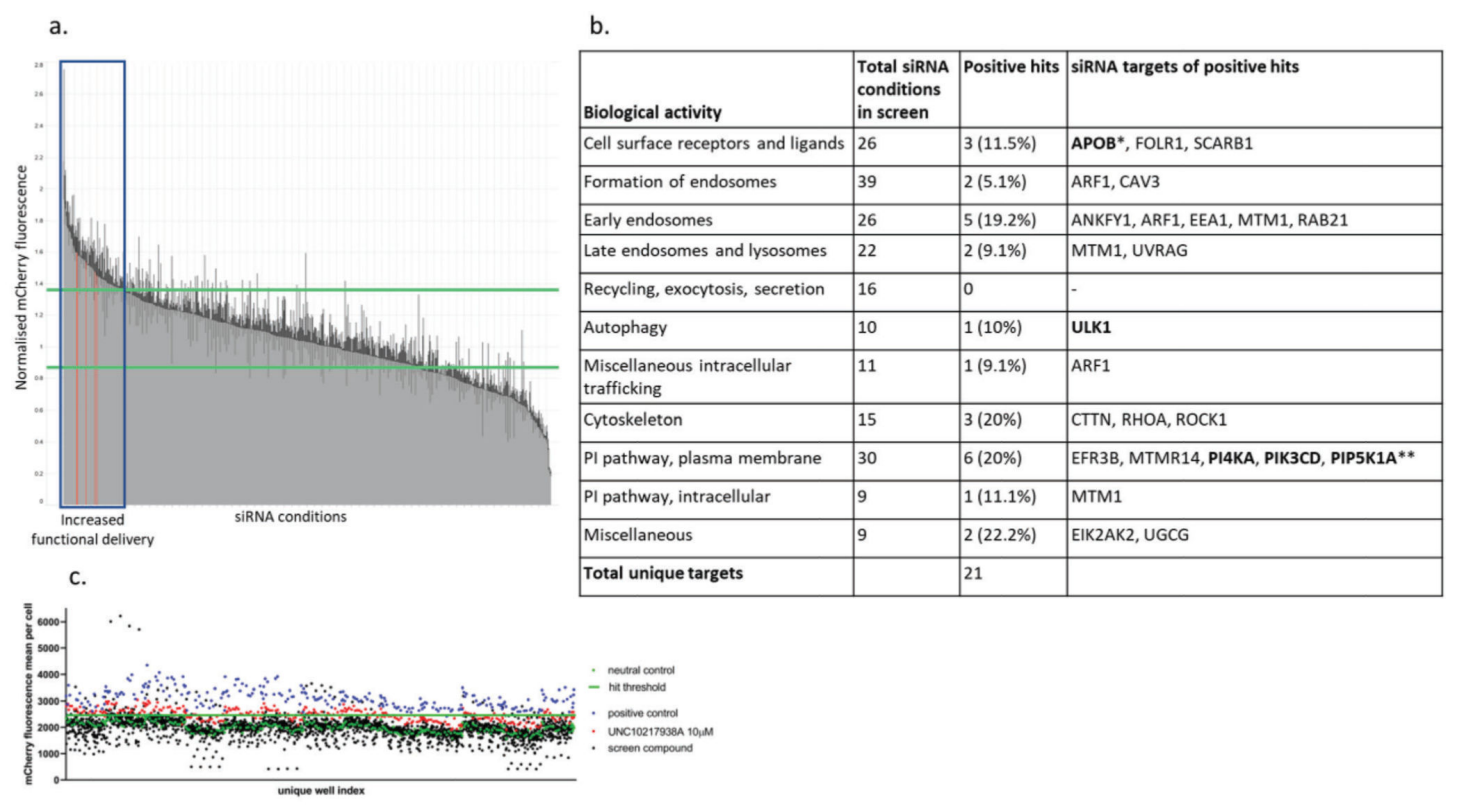
siRNA screening identifies targets throughout the endosomal system, which affects the functional delivery of mRNA by LNP. a) Results of the siRNA screen for mRNA-LNP functional delivery, targeting proteins of the endosomal and phosphatidylinositol systems. Results were normalized to a neutral control siRNA, with “neutral” performance in the screen defined as the 3 standard deviations surrounding the mean of this neutral control siRNA (range defined by green lines), and siRNA treatment above this range classified as “increasing functional delivery” (blue box). The positive control was cells transfected with neutral siRNA and treated with double the LNP concentration (red bars). For each targeting siRNA, four concentrations were tested (1, 5, 10, 25 nm; gray bars). Bars show mean and standard deviation of each treatment condition. b) Distribution of biological activity of siRNA targets tested in the siRNA screen. Compounds against this target were also positive hit in compound screen (bold). *Expression in NCI-H358 was not confirmed by RT-qPCR. **Two active siRNA sequences were identified, while all others were used as pools of four siRNA. c) A screen for mRNA-LNP functional delivery was performed with compounds predicted to target proteins previously identified by siRNA screening (black). DMSO was used as a neutral control (green); and wells treated with endosomal escape enhancer UNC10217938A (red) or with double the LNP concentration per well were used as a positive control (blue).

Phosphoinositides are known to regulate endocytosis^[11]^ and so a screen of 178 siRNA conditions targeting the phosphatidylinositol pathway, plasma membrane receptors, endosomal and intracellular trafficking systems was performed in NCI-H358 cells (Figure 2a). Targets were considered to improve the functional delivery of mRNA-LNP if mCherry fluorescence was at least three standard deviations from the mean of a neutral control siRNA that did not engage the RNA-induced silencing complex (RISC-free). These screens identified 22 siRNA treatments (covering 21 genes), which repeatably increased functional delivery (Figure 2b).

A series of 233 compounds were identified, which bound to some of the protein targets identified in the siRNA functional delivery screens (Figure 2c). The compounds were then tested in a modified functional delivery screen, where cells were seeded without siRNA transfection, and then a test compound and mRNA-LNP were added to cells at the same time, incubated for 24 h, and imaged as per Figure 1d. This functional delivery assay identified 14 compounds that were active in increasing mRNA functional delivery, targeting APOB, PI4KA, PIP5K1A, PIK3CD, PIK3CG, and ULK1 (Figure S1, Supporting Information). A selection of previously published compounds (including EIPA, Dynasore, Pitstop2, and UNC10217938A)^[12]^ was found to have minimal effect on functional delivery at the maximum screening concentration (10 μm) (Figure S2, Supporting Information).

Thus far, phenotypic screening had identified 22 siRNA and 14 compound treatments, which increased functional delivery, but with little insight into the mechanisms by which they do this. A series of mechanistic assays were performed on cells treated with these siRNA and compounds, along with 70 siRNA and compound conditions with presumed biological activity but no effect on mRNA-LNP functional delivery (defined as performing within three standard deviations of the neutral control in the above siRNA or compound screens). For downstream analysis, treatments were classified either as increasing functional delivery or as having no effect.

Three mechanistic assays were chosen to characterize the endocytic pathways of treated cells: endocytosis of fluorescently labeled mRNA in LNP; fluorescently labeled transferrin, which binds to the transferrin receptor and is internalized by clathrin-mediated endocytosis; and fluorescently labeled 70 kDa dextran to detect nonselective fluid-phase endocytosis (including macropinocytosis). The resulting confocal microscopy images from these experiments were analyzed as mean fluorescence intensity per cell, and total number of fluorescent spots per cell.

**Figure 3:**
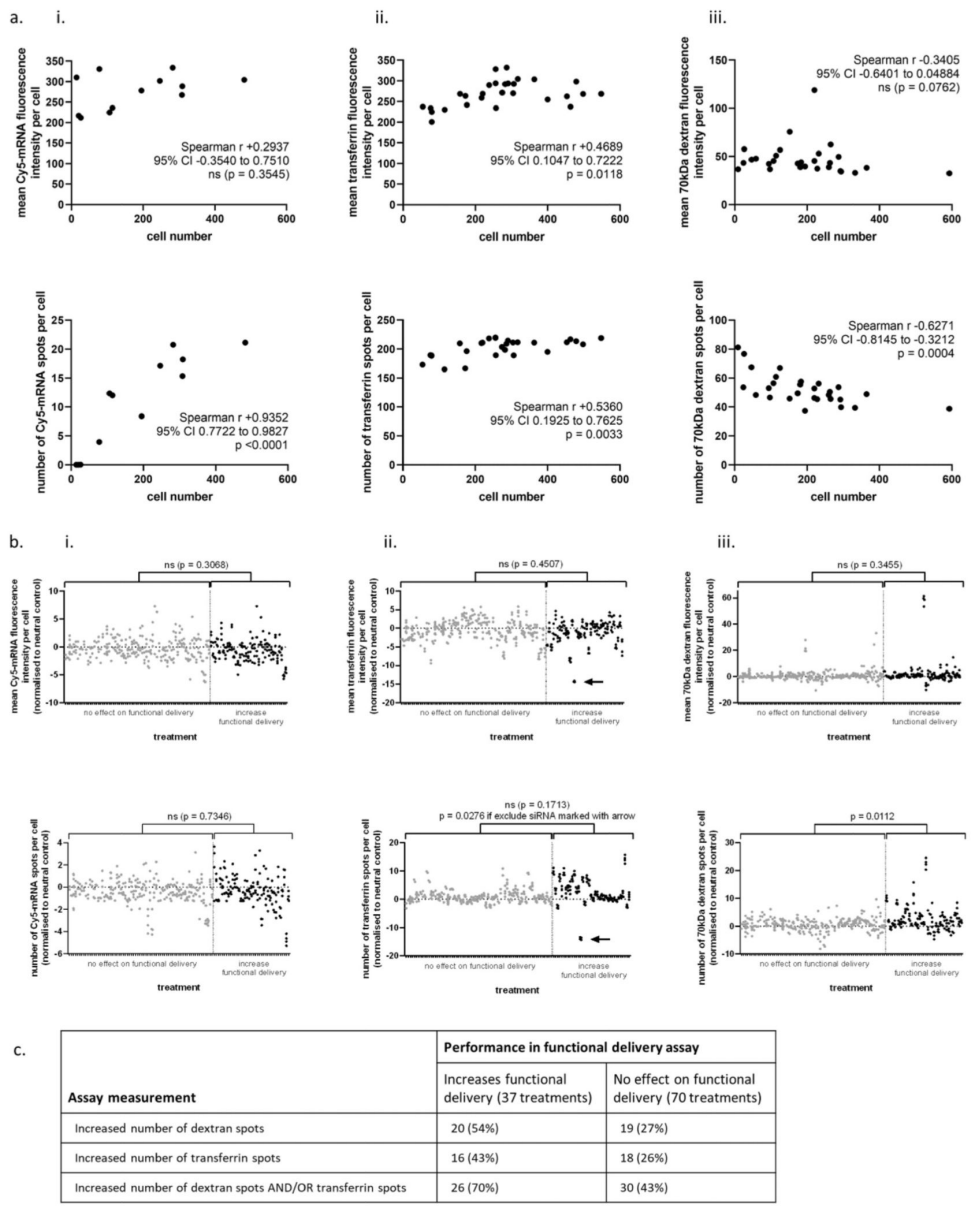
Standard analyses used to describe mechanisms for mRNA–LNP functional delivery. a) Cell number variation in standard analyses of the (i) Cy5-mRNA in LNP, (ii) transferrin, and (iii) 70 kDa dextran uptake assays. Spearman’s correlation with two-tailed *P*-value. b) Standard image analysis of fluorescent marker uptake assays, separated by performance in the functional delivery assay. Each technical replicate was normalized by robust Z-score to the siRNA neutral control (RISC-free siRNA) or compound neutral control (DMSO) as appropriate. Each spot represents one technical replicate, with 2–4 technical replicates per treatment (siRNA or compound) condition. Unpaired *t*-test with Welch’s correction, two-tailed *P*-value of positive vs neutral treatment populations; ns = not significant (*p* > 0.05). Black arrows mark the same outlier for siRNA treatment in both graphs. c) Identification of phenotypic outliers in the transferrin and dextran uptake assays, as a method of identifying positive performance in the functional delivery assay. A treatment was considered to increase dextran or transferrin spots if the treatment achieved a robust Z-score of >3 in at least one technical replicate. This shows that there is a weak correlation between performance in the transferrin or dextran assays and functional delivery.

We observed that most of these endocytic measures were somewhat sensitive to cell density (Figure 3a). The siRNA and compound treatments had, however, been classified as increasing functional delivery independently of their effects on cell number, so any effects on LNP performance should be apparent regardless of cell culture density.

We compared two groups of treatments: those with no effect on mRNA-LNP functional delivery or treatments that increased functional delivery. Statistically significant differences were only found when spot counts in the transferrin and dextran endocytosis assays were compared. Treatments that made LNP perform better increased the number of spots observed in both assays, while LNP uptake was unaffected (Figure 3b). To evaluate this another way, we used a robust Z-score threshold of >3 to identify treatments within each group that were quite different from the neutral control. These results were tabulated to evaluate whether these changes could robustly describe differences between the treatments that did or did not improve functional delivery (Figure 3c).

Overall, we found that the traditional analyses of mRNA-LNP, transferrin, and 70 kDa dextran uptake were not sufficient to describe meaningful phenotypic differences that our panel of siRNA and compound treatment conditions were producing. We therefore sought to explore these treatment phenotypes using an unbiased, data driven, multivariate approach. Using machine learning we tested the hypothesis that there were more predictive phenotypic features within these image sets.

**Figure 4:**
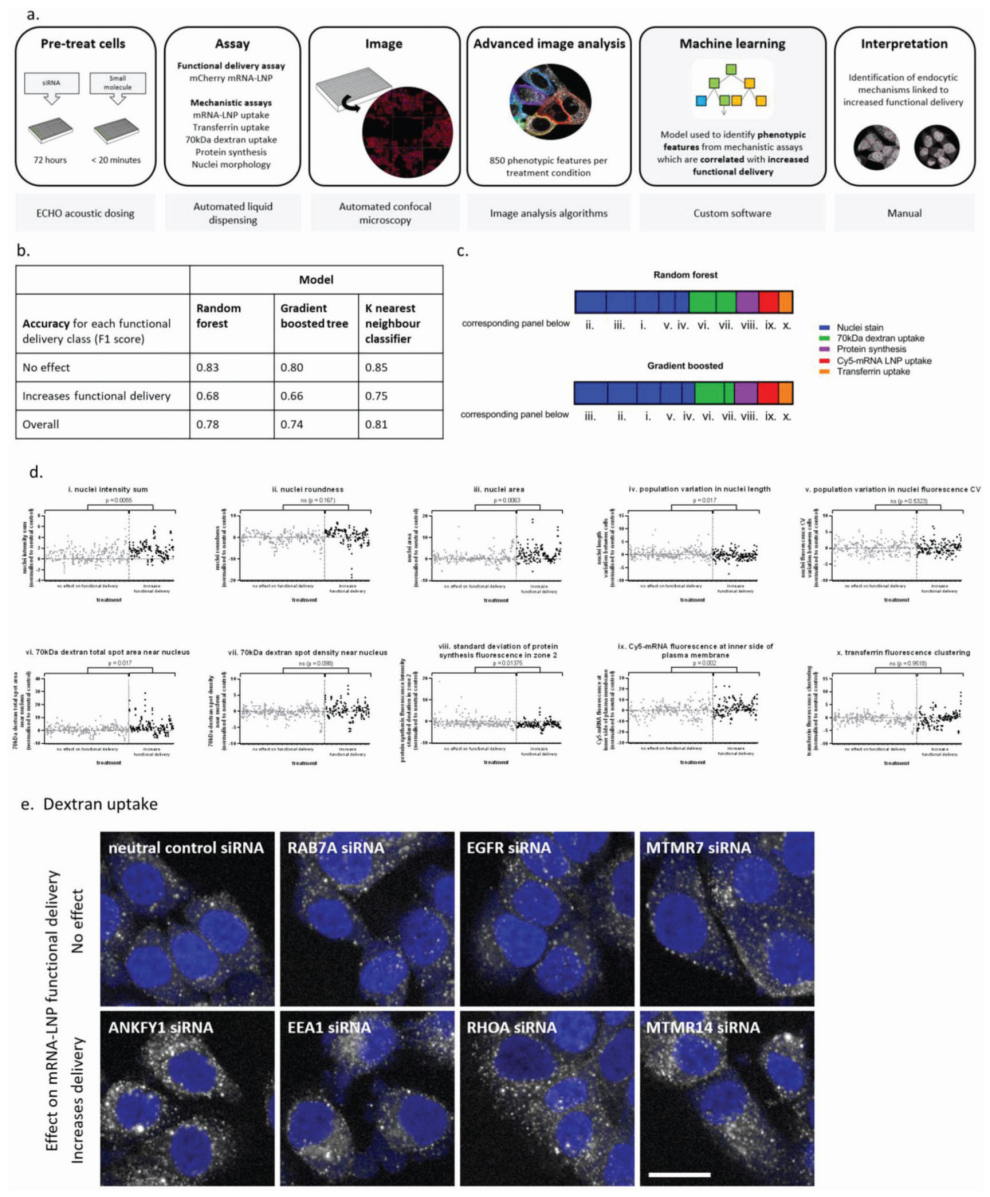
Machine learning to identify cell phenotypic features important for productive delivery. a) Schematic of data preparation and machine learning workflow for ACE-ID. b) Machine learning models can differentiate between different classes of productive delivery performance, utilizing only 10 phenotypic features of endosomal/macropinosomal and protein synthesis activity. Accuracy as measured by F1 score. c) The weighted importance of each of the 10 features utilized by random forest and gradient boosted models described in (b). d) Productive delivery class distribution of selected top phenotypic features that were identified by machine learning models, with normalization control marked with dotted line. Features were derived from (i–v) nuclear stain, (vi, vii) 70 kDa dextran endocytosis assay, (viii) protein synthesis assay, (ix) endocytosis of Cy5-mRNA in LNP, and (x) transferrin endocytosis. Unpaired *t*-test with Welch’s correction, two-tailed *P*-value of positive versus neutral treatment populations with correction for multiple testing (Benjamini and Hochberg’s false discovery rate); ns = not significant (*p* > 0.05). e) Uptake of 70 kDa dextran (white) in selected siRNA-treated cells (nuclei, blue). Scale bar: 20 μm.

A total of 850 phenotypic features for each siRNA- or compound-treated cell population were extracted using advanced image analysis. These features encompassed cell number, cell and nuclear morphology, and the intensity and texture of the fluorescence of the assay marker within each cell and across the cell population. The robust Z-scores for these measurements were normalized to the neutral control in each assay (Figure 4a). In addition to the three endocytosis assays described above, this step also included a fourth fluorescent imaging assay to measure the rate/extent of protein synthesis.

Machine learning was then used to interrogate the normalized phenotypic data set, with the goal of identifying which phenotypic features were most useful in predicting whether a treatment would improve LNP functional delivery. Models were initially generated with all 850 available phenotypic features (Figure S3, Supporting Information), however we found that we could achieve similar accuracies and improve interpretability using just ten of these features. These ten features were selected using recursive feature elimination with a Gradient Boosted Tree model. Three models (Random Forest, Gradient Boosted Tree, and K-Nearest Neighbor) were subsequently trained using just these features (Figure 4b). The produced models with an overall accuracy (F1 score) of 74–81%, and class-specific accuracy of 66–75% in identifying treatments which increase functional delivery. Feature weightings were extracted from the Random Forest and Gradient Boosted Tree models, with both utilizing the ten features in a very similar pattern. Both models weighted nuclei stain features heavily (Figure 4c), indicating that siRNA and compound treatments that increase functional delivery of mRNA-LNP generally cause the nuclei of cells to become larger, more rounded and overall more intensely stained by NuclearMask blue (Figure 4d(i–v)).

The next-heaviest weighted features in the models were from the 70 kDa dextran uptake assay. These were both measures of the fluorescent puncta of 70 kDa dextran in the cytosolic zone closest to the nucleus, which was defined as spanning from the nuclear boundary to 50% of the width of the cytoplasm. Treatments with positive effects on functional delivery increased both the total area of 70 kDa dextran spots and spot density, suggesting that movement of vesicles into this area of the cell was a strong indicator of improved functional delivery.

The lowest-ranked feature in both the Random Forest and Gradient Boosted Tree models was from the transferrin assay, which was the clustering of transferrin fluorescence intensity. On its own, this feature of transferrin distribution is not statistically significantly different between treatments that have positive and neutral effects on functional delivery, indicating that this is not a useful stand-alone feature to predict functional delivery performance, but that it is valuable when integrated into a multivariate model.

**Figure 5:**
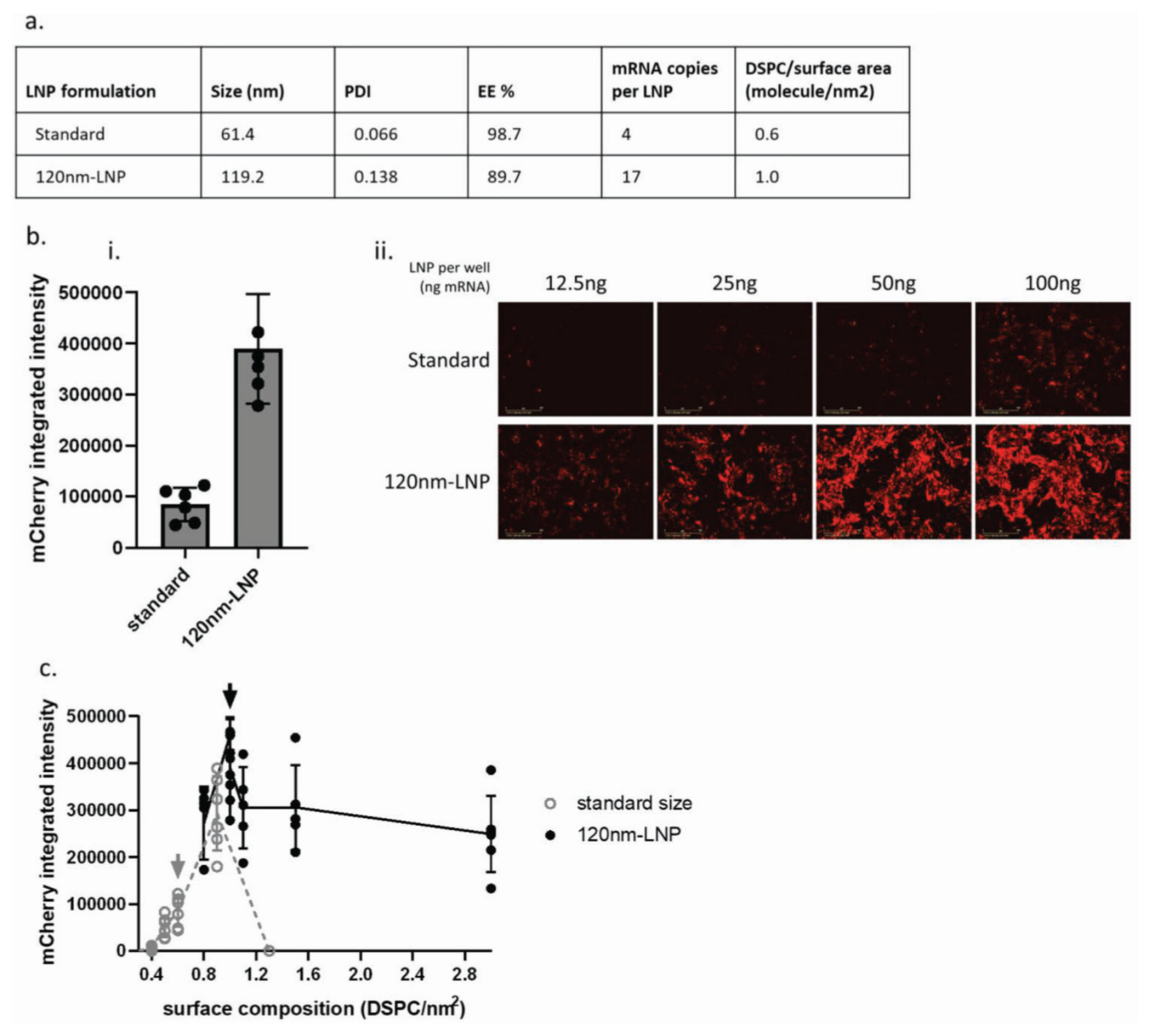
Rational design of LNP for improved mRNA delivery in vitro. a) LNPs were formulated to the standard and 120 nm-LNP size, representative data of LNP formulated with Fluc mRNA. b) In NCI-H358 cells, the 120 nm-LNP achieved higher protein production, (i) as quantified by integrated fluorescence intensity in cells treated with 25 ng per well (0.5 μg mL^−1^), mean and standard deviation, *n* = 5; and (ii) example widefield images. c) The surface composition of LNP was modified while keeping size constant. Cells were incubated with 25 ng per well of each LNP for 24 h. mCherry integrated fluorescence intensity was measured by Incucyte in live cells. Lines connect mean of each series of surface compositions at a given size, error bars show standard deviation of *n* = 3–5. Gray arrow, size, and surface composition of standard formulation. Black arrow, size, and surface composition of 120 nm-LNP formulation.

As multiple models identified that changes in features of fluid-phase endocytosis were correlated with improved functional delivery of mRNA, we sought to test whether macropinocytosis may be an efficient way to deliver mRNA-LNP to NCI-H358 cells. We therefore adjusted the formulation process to generate larger-diameter LNP (“120 nm-LNP”) with the same overall lipid composition and pKa to our standard LNP composition (“standard”) (Figure 5a; Figure S4, Supporting Information). These particles were designed to be slightly too large to fit into the internal diameter of canonical clathrin-coated vesicles, which accept particles up to 80—100 nm in diameter,^[13,14]^ and we hypothesized that they would be predominantly internalized by macropinocytosis. Indeed, although uptake of these particles was very similar to our standard LNP (Figure S5, Supporting Information), the 120 nm-LNP delivered mCherry mRNA to NCI-H358 cells more efficiently (Figure 5b,c), with a 6.3-fold improvement in protein expression.

To confirm that the 120 nm-LNP were being internalized by a nonselective fluid-phase mechanism, we systematically altered the surface composition of LNP while maintaining the size difference between the standard and 120 nm-LNP formulations (70 and 120 nm, respectively; Figure 5c; Figure S4d, Supporting Information). This was achieved by varying the relative percentages of DSPC and cholesterol, while keeping MC3 and PEG-lipid percentages constant. This would be expected to modify the amount of DSPC and cholesterol exposed on the LNP surface.^[15,16]^ The smaller standard LNP showed a marked sensitivity to the surface composition of the particles, with an optimal surface composition that elicits the most efficient functional delivery. For standard-sized LNP, functional delivery was improved by moderately increasing DSPC from 10% to 12%, which has also been observed elsewhere.^[17]^ This suggests these particles being internalized by a receptor-mediated mechanism, with a binding interaction, which is sensitive to changes in the particle surface. The functional delivery efficiency of the larger-sized, 120 nm-LNP was relatively insensitive to modifications to the particle surface composition, indicative of a nonselective endocytic mechanism consistent with macropinocytosis.

**Figure 6:**
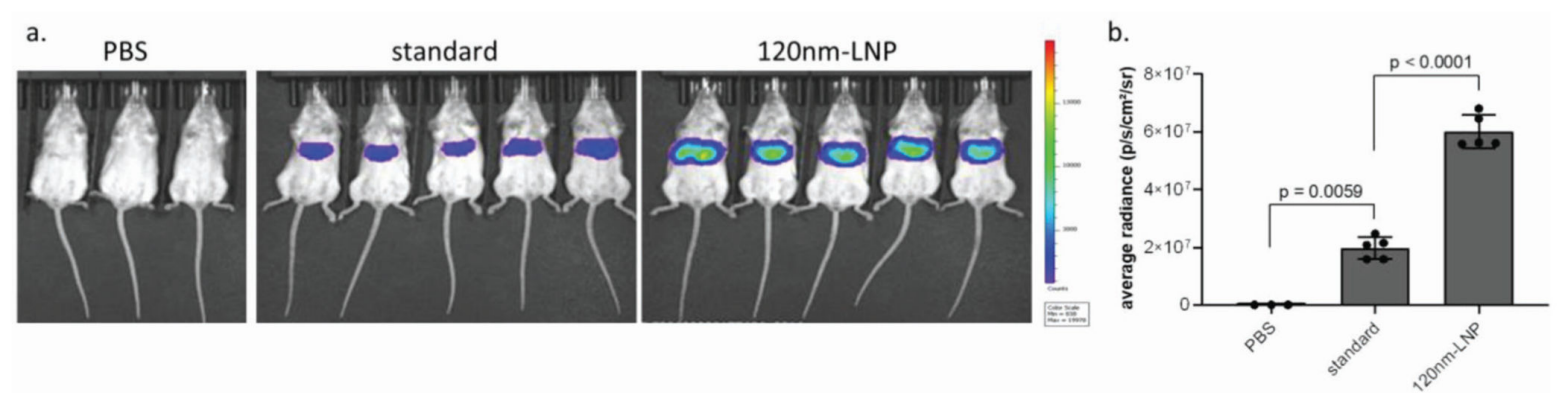
Larger LNP improved mRNA delivery in vivo. Mice were dosed intravenously with 0.25 mg kg^−1^ standard or 120 nm-LNP containing mRNA encoding for luciferase. 6 h after dosing, luciferase expression was assessed using a) an IVIS spectrum imager and b) average radiancy was measured, showing that 120 nm-LNP resulted in more functional delivery to the mouse liver. One-way ANOVA with Tukey’s multiple comparisons test, *n* = 3–5.

Finally, mice were dosed intravenously with standard or 120 nm-LNP formulations containing mRNA encoding firefly luciferase (Fluc), such that each mouse received the same dose of mRNA. Six hours after dosing, Fluc activity was predominantly detected in the liver for all LNP formulations, with threefold more luminescence activity detected for the 120 nm-LNP than the standard formulation (Figure 6).

## 3 Discussion

**Figure 7:**
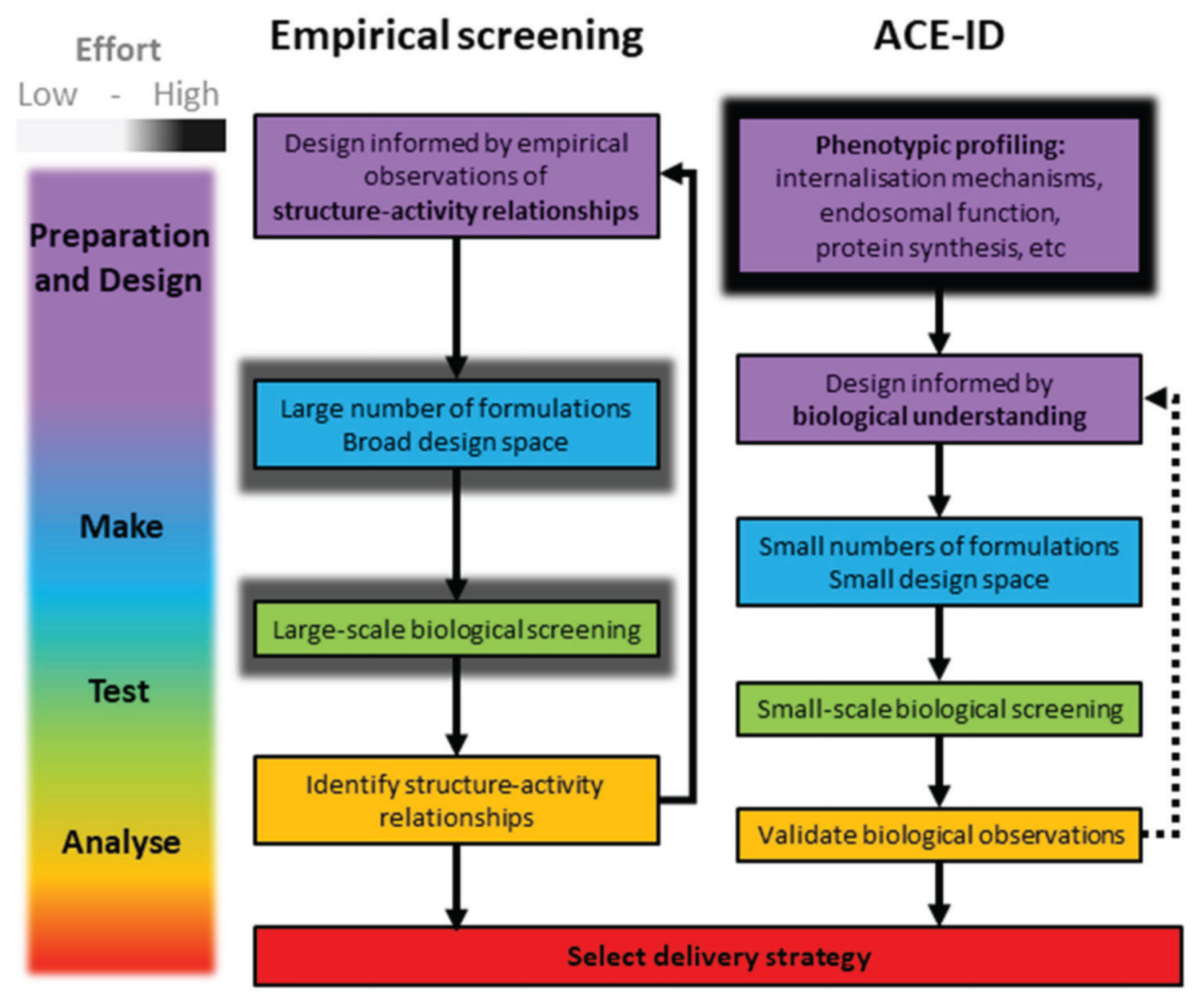
Comparison of workflows for the development of nanomedicine delivery strategies. Empirical screening of delivery systems relies on iterative cycles of high-throughput formulation testing. In the ACE-ID pipeline, effort is front-loaded into understanding the cellular target of the nanomedicine, which then informs the generation and testing of a small number of formulations. Solid arrows, required workflow; dotted arrow, discretionary workflow path.

Empirical screening approaches centered around the delivery materials have identified increasingly potent reagents for the delivery of nucleic acids. Examples include polyamine core lipidoids (C12-200,^[18]^ Ckk-E12,^[19]^ 503013),^[20]^ and DLinDMA derivative lipids (DLin-MC3-DMA,^[21]^ L319,^[22]^ and Lipid 5^[7]^ published by Benenato and co-workers). When compared to small molecule drugs, which are designed to treat known disease mechanisms, we know relatively little about how nucleic acid delivery materials function at an intracellular molecular level. Inspired by the target biology-focused modern drug discovery paradigm, we designed a biology-centered process to rationally design a nanomedicine delivery system. We applied this workflow to optimize the clinically approved MC3-LNP delivery system termed ACE-ID (Figure 7). The advantage of this approach is that delivery systems can be optimized specifically for a particular route of entry, reducing the likelihood of side effects and improving efficacy.

First, we sought to understand the biology relevant for improved mRNA-LNP functional delivery, by treating a lung cancer cell line, NCI-H358, with 178 siRNA and 233 small molecule compounds chosen to perturb biological processes hypothesized to be relevant for intracellular delivery. It is well-established that there are weak correlations between in vitro and in vivo performance of LNPs,^[23]^ however strong evidence is available that endosomal entrapment is a bottleneck.^[6]^ Therefore, to demonstrate the ACE-ID workflow, we utilized a cell line, which has been identified as exhibiting low transfection efficiencies due to a high level of endosomal entrapment,^[9]^ as it has previously been shown that mRNA-LNP delivery can be improved by compounds that enable endosomal escape.^[12]^

The effect of these perturbations on the functional delivery of LNP loaded with mRNA encoding mCherry was then examined using an image-based assay. 22 siRNA treatments were found to enhance functional LNP delivery, revealing clues about what mechanisms could be targeted to improve delivery. For example, consistent with a previous observation that siRNA-containing LNP were released from EEA1-negative, RAB5-positive endosomes in HeLa cells,^[24]^ we found that knockdown of EEA1 increased functional delivery of mRNA, and knockdown of RAB5C (although not RAB5A or RAB5B) reduced functional delivery.

To understand the mechanisms of improved functional delivery further we performed three well-established in vitro assays for profiling endocytic activity of cells. These were the endocytosis of fluorescently labeled mRNA formulated into LNP, transferrin, and 70 kDa dextran. These measurements were not very useful in understanding the mechanisms by which our treatments affected cell phenotype, in part because performance in all three assays was affected by cell density (Figure 3a). Our data show that it is vital to understand and account for how an assay read-out can vary even with relatively minor changes in cell density.^[25–27]^

A second important observation from the standard endocytosis assays used in the present work is that they often did not show statistically significant differences between treatments which did and did not affect functional delivery (Figure 3b). Including biologically active siRNA and compounds that do not affect functional delivery in these assays enabled us to see how wide the phenotypic variation can be without having any meaningful effect on functional delivery, which demonstrates how little utility these measurements have in explaining the mechanism by which a given treatment increases functional delivery of mRNA-LNP. Indeed, approximately half of the treatment conditions identified as increasing clathrin-mediated or fluid-phase endocytic activity by either the transferrin or 70 kDa dextran assays actually had no effect in the functional delivery assay (Figure 3c). Conversely, 30% of the treatments that we knew to increase functional delivery were not identified as increasing endocytic activity in either of these mechanistic assays.

As these traditional mechanistic assays failed to identify cell phenotypes which correlated with functional delivery, we expanded our dataset to include a measure of the rate and subcellular distribution of protein synthesis. Images from the three endocytic assays (mRNA-LNP, transferrin, and 70 kDa dextran up-take) and a protein synthesis assay, along with the nuclei stains utilized in every image set, were then analyzed.

Advanced image analysis algorithms were used to extract 850 phenotypic features from each treatment condition to generate in a rich data set of information about how specific siRNA/small molecule treatments may be affecting nanoparticle delivery. Aiming to identify the specific phenotypic features correlating with siRNA treatments that improved LNP delivery, we employed machine learning to generate unbiased multiparametric correlations. Multiple machine learning models yielded similar insights into the phenotypes that correlated best with improved productive delivery, demonstrating the robustness of these findings. Models utilizing just 10 features reached similar conclusions to those which utilized all 850 features: the most important information on cellular phenotype is encoded in, rather surprisingly, the nuclei stain, and also two readouts from the fluid-phase endocytosis marker 70 kDa dextran, identifying spot proximity to the nucleus as an important feature. The relationship between endosomal trafficking toward the nucleus and mRNA-LNP functional delivery has previously been observed by comparison across cell types.^[9]^ On closer examination of our images, the spot size indicated that the marker was primarily localized in relatively large vesicles, similar in size to macropinosomes.^[28]^ It has been reported before that macropinosomes traffic to an area near the nucleus but do not necessarily fuse with late endosomes/lysosomes.^[29,30]^ In further support of this, siRNA knockdown of several macropinosome-related proteins was found to increase mRNA-LNP functional delivery, perhaps by further slowing macropinosome maturation/trafficking: ANKFY1 (Rabankyrin-5; which has previously been shown to be important for LNP trafficking),^[6,31–33]^ PI3 kinases,^[34]^ RHOA,^[35]^ and its effector ROCK1. Conversely, we observed that knockdown of macropinocytosis regulators CDC42 and RAC1 were seen to slightly decrease functional delivery. Interestingly, these data are in agreement with previous studies suggesting that siRNA-LNP formulated with different cationic lipids and siRNA cargo also entered HeLa cells and fibroblasts via this pathway, perhaps even inducing macropinocytosis.^[6,36]^ It has also recently been shown that siRNA delivery by Lipofectamine could be improved by stimulating macropinocytosis and inducing membrane lysis using a peptide conjugate of SN21 and LK15.^[37]^

Because the observations of fluid-phase endocytosis were consistent with these compartments being macropinosomes, we designed an LNP that would predominantly be internalized by macropinocytosis. We found that, unlike the standard 70 nm-LNP, the mRNA delivery efficiency of the larger 120 nm-LNP formulations was not dependent on particle surface composition, consistent with a nonreceptor-mediated endocytic mechanism like macropinocytosis. This is consistent with other recent work, whereby large LNPs made by an automatic pipetting system were seen to be internalized by a mechanism consistent with macropinocytosis.^[38]^ Indeed, other studies have also indicated that larger LNP sizes are capable, and may be favorable, for mRNA delivery.^[16,39]^ This is in contrast to siRNA, where smaller LNP (similar to our “standard” formulation) have been found to be optimal.^[40–43]^ This may be due to the loading capacity of LNP being able to accommodate fewer copies of mRNA molecules, which can be 20- to 50-fold larger than siRNA. When LNP size is increased, the loading capacity for mRNA is also increased, so one LNP particle can deliver more mRNA copies to cells.^[38,44]^

Interestingly, although labeled mRNA encapsulated in both standard and 120 nm-LNP formulations was observed to be taken into cells at a similar level, the 120 nm-LNP resulted in more efficient functional delivery of mRNA in NCI-H358 cell cultures and also in vivo, where expression is primarily located in the liver. By dosing matched quantities of mRNA, we effectively dosed fewer 120 nm-LNP particles compared to the standard formulation, due to 120 nm-LNP having more mRNA loading capacity. Therefore the improved functional delivery by 120 nm-LNP may be due to a combination of a favorable LNP composition and conducive intracellular trafficking route, which enables more efficient delivery of mRNA for translation into protein. While it may seem unusual that a lung cancer cell line was able to predict liver delivery, we believe this supports the utility of characterizing a large range of cellular perturbations in order to select the most generalizable features for delivery. When the ACE-ID workflow is applied to a different in vitro cell model, we may find that the optimal LNP formulation is different and selective for a different cell type in vitro and in vivo.

Moving toward a biology-centered approach will enable the rational design of strategies for nucleic acid delivery systems. This is an important step for the development of safer, more effective delivery systems for nucleic acid delivery. Previously, this has been difficult due to the sheer complexity of the biology of intracellular delivery. We have shown that strategic use of laboratory automation, image analysis, and machine learning in the ACE-ID workflow can be leveraged to identify specific mechanisms to guide the design of nanoparticle-based intracellular delivery systems, and that this can be used to yield improved nucleic acid drug delivery in vivo. Furthermore, this shows a target biology-based modern drug discovery process can be used to optimize delivery systems for intracellular delivery. We believe the application of ACE-ID type approaches in the field of intracellular drug delivery will ultimately accelerate the clinical progression of safe and effective nucleic acid-based therapeutics.

## 4 Experimental Section

### Cell Culture and siRNA Transfection

For cell phenotypic characterization assays (LNP functional delivery screening, endocytosis, and protein synthesis assays), NCI-H358 cells (ATCC) were grown in phenol red-free RPMI-1640 (Sigma), supplemented with 10% v/v foetal calf serum (Life Technologies) and 2 mm GlutaMAX-I (Life Technologies) (growth media), in a humidified 5% CO_2_ atmosphere at 37 °C. When testing LNP formulations, NCI-H358 cells were grown either as above, or with RPMI-1640 (Gibco, ThermoFisher) supplemented with 10% heat-inactivated FBS (Gibco, ThermoFisher) and 2 mm GlutaMAX (Life Technologies).

When required, cells were reverse-transfected by pre–incubating 1–25 nm siRNA with Lipofectamine RNAiMAX (Invitrogen, cat. 13778150) diluted in Optimem (Life Technologies, cat. 31985062) in CellCarrier Ultra 384-well plates (Perkin Elmer, cat. 6057308). NCI-H358 cells were then seeded and incubated for 72 h in a humidified 5% CO2 atmosphere at 37 °C.

Alternatively, untransfected NCI-H358 cells were seeded in CellCarrier Ultra 384-well plates (for confocal microscopy) or poly-d-lysine coated black clear-bottom 384-well plates (Greiner; for widefield microscopy) and incubated for 24 h before treatment with compounds, LNP and/or endocytosis markers.

### Lipid Nanoparticle Preparation

LNPs were prepared as described previously.^[16]^ DLin-MC3-DMA, DSPC, cholesterol, and DMG-PEG2000 were dissolved in ethanol and used at a 50:10:38.5:1.5 molar ratio for the standard and 120 nm-LNP formulations. The lipid composition of LNPs with varied surface compositions (results shown in Figure 5) is shown in Figure S4c of the Supporting Information. Fluc mRNA or mCherry mRNA (Trilink cleancap, 5MoU), or GFP Cy5-mRNA (Trilink, 5 meC, Ψ) were dissolved in 50 mM citrate solution (pH 3). The lipids and mRNA were mixed in a Nanoassemblr microfluidic device (Precision NanoSystems) with weight ratio of lipids:mRNA 20:1. For standard LNP, a volume ratio of lipids:mRNA 1:3 was used together with a mixing flow rate 12 mL min^−1^. For 120 nm-LNPs, both volume ratio and flow rate were adjusted to meet size criteria. For all the formulations, the resultant LNPs were dialyzed in PBS overnight at 4 °C before use.

### Lipid Nanoparticle Characterization

The hydrodynamic 𝜁 size of all LNP was measured using a Malvern Zetasizer at a back scattering angle of 173 °C. LNP were diluted 100× in PBS before Zetasizer measurement and the resultant hydrodynamic intensity size and PDI were recorded. The volume-weighted particle size distribution was exported for simulation of DSPC surface of LNP. Encapsulation efficiency of mRNA by LNP was detected by Invitrogen Ribogreen RNA assay kit (Thermo Fisher Scientific).

Size distribution of LNP was analyzed on a NanoSight NTA 3.0 instrument (Malvern-Panalytics) using a red laser for light scattering acquisition. LNP were diluted 1000–4000-fold in sterile PBS (0.02 μm filter) to obtain 50–200 particles per frame in the field of view. For measurements, each sample was recorded for five times and 60 s per time with a syringe pump infusion at 50 μL min^−1^. The camera level was set 11 and the analysis detector threshold was 5. The acquisition and data analysis were performed using Nanosight NTA 3.0 software (Malvern-Panalytics). Size distribution was plotted as size versus normalized particle concentration (particle/mL) using Prism (version 8, GraphPad) in order to compare the size distribution of standard LNP and 120 nm-LNP.

The surface pKa value of LNP were determined by using TNS assay (2-(p-toluidinyl)naphthalene-6-sulfonic acid, sodium salt, Sigma-Aldrich).^[21]^ The assay was performed in a black 96-well plate by titrating buffers (10 mm sodium acetate or phosphate and 150 nm sodium chloride) to pH value varying by 0.5 from 4.0 to 9.0. The LNP and TNS were diluted into these buffers at a final concentration of 25 and 6 μm. Fluorescence intensity was collected on a fluorescence plate reader (Envision, Perkin Elmer) using excitation and emission of 323 and 435 nm. Data were analyzed using GraphPad Prism assuming maximal fluorescence corresponding to 100% protonation and minimal fluorescence corresponding to 0% protonation. pKa was calculated as the pH corresponding to 50% protonation as fitted using nonlinear sigmoidal dose–response (variable slope).

For experiments comparing the surface composition and size of LNP, the DSPC surface of LNP was derived from their volume-weighted particle size distribution as previously described.^[16]^ Various molar ratios of DLin-MC3-DMA, DSPC, cholesterol, and DMG-PEG2000 were used, as listed in Figure S4D of the Supporting Information, and then combined with mRNA by microfluidicmixing and the resulting LNPs were characterized as above.

For functional delivery screening, LNP were stored in 10% glycerol in PBS at −20 °C and thawed immediately before use. Performance after freeze/thawing was verified by comparing concentration–response ranges (0–100 ng per well) of fresh LNP (less than one week after formulation) to the same LNP batch that had been frozen overnight, and a different LNP batch that had been frozen for 3 months. For assays which compared the functional delivery of 120 nm-LNP in vitro and in vivo, formulations were stored at 4 °C in PBS and used within one week.

### siRNA and Compound Selection

Two siRNA libraries were utilized in screening. One contained 103 targets involved in uptake and intracellular trafficking pathways (siGenome, Dharmacon), while the other contained 58 kinases, phosphatases, and regulatory subunits of the PI pathway (ON-TARGETplus, Dharmacon). Pools of four siRNA were utilized for to knockdown majority of targets during screening and hit characterization. RISC-free (siGenome, Dharmacon) was used as a neutral control treatment. A list of the siRNA used can be found in Section S2 of the Supporting Information.

Compounds were identified that were annotated to bind to a selection of siRNA targets. All compounds with activity annotations for 34 siRNA targets (21 repeatable targets, plus 13 nonrepeating siRNA conditions) identified to increase mRNA-LNP functional delivery were collected from the internal database.^[45]^ The highest reported activity was retained for compounds with multiple measurements. Applying a threshold of more than 100 nm activity on the target provided 5205 compounds for 10 targets. Table S1 of the Supporting Information provides the distribution of annotation compounds for these targets. Chemical clustering was performed using extended-connectivity fingerprints with a width of 6 bonds (ECFP6)^[45]^ to select potent and structurally diverse subset for each target with more than 100 compounds. Additionally, compounds with several target annotations yet selective for those of interest and purity above 80% were prioritized. A total of 200 compounds were identified for testing, with multiple synthesis batches available for some. A total of 233 compounds were tested in 3-point (10 *μ*m, 1 *μ*m, 0.1 *μ*m) concentration–response screens. Compounds that increased functional delivery more than three standard deviations above the mean of the DMSO control were then validated in a 10-point (10 *μ*m –0.3 nm) concentration–response assay.

Other known compounds were included in functional delivery screens and mechanistic assays: UNC10217938A, UNC4267, and UNC2383^[12]^ up to 10 *μ*m; and 5-(N-ethyl-N-isopropyl)amiloride (EIPA; Sigma, cat. A3085-25MG), Dynasore (Abcam, cat. ab120192), and Pitstop2 (Abcam, cat. ab120687) up to 40 *μ*m. MG-132 (Sigma, cat. 474791-1MG) and cyclohexamide (Sigma, cat. 239765-1ML) were included as controls for selected mechanistic assays. All compounds were dissolved in DMSO.

### mRNA-LNP Functional Delivery Screening Assays Using Automated Confocal Microscopy

Cells were reverse-transfected in 384-well plates with 1, 5, 10, and 25 nmol of each siRNA and incubated for 72 h. In the case of cells treated with compounds, compounds dosing was performed by Echo acoustic dispenser (model 555, Labcyte Inc.) to adherent cells immediately before addition of LNP. LNP containing mRNA encoding mCherry were diluted in 10 μL per well growth media (containing FCS) and applied to cells, dosing cells with 0.4 μg mL^−1^ (20 ng per well in a 384-well plate) mRNA. Cells and LNP were then incubated for 24 h, then fixed with 4% paraformaldehyde (PFA) in PBS and stained with Hoechst 33342 (Invitrogen, cat. H3570).

Samples were imaged on a Yokogawa CV8000 automated confocal microscope, using a 20× air objective (numerical aperture 0.75) at a single z-plane. Functional delivery of mCherry mRNA was determined by the mean cellular fluorescence of mCherry protein per cell.

Automated image analysis was performed as described below. For siRNA screens, where cell density changes occurred before addition of LNP, a normalization strategy was developed to account for the relationship between cell density and mRNA functional delivery (see Section 2 and Figure 1c). For each assay, wells were seeded with a range of cell densities (transfected with a neutral control siRNA, RISC-free), which allowed a one-phase decay curve to be fitted using Prism (version 8, GraphPad) that represented the relationship between cell number and functional delivery. Sample results were normalized using this curve (Tibco Spotfire), with deviation representing an increase or decrease of mCherry expression, accounting for changes in cell density. For compound screens, no normalization was required, as cell density was equal between all wells at the time of LNP addition.

siRNA or compound treatments were classified as increasing functional delivery if the mean of the technical replicates crossed the hit threshold, defined as three standard deviations above the mCherry mean fluorescence (raw or normalized) of the neutral control treatment (RISC-free siRNA or DMSO, for siRNA and compound screens, respectively).

### Endocytosis Assays

For all endocytosis assays, NCI-H358 cells were either transfected with siRNA 72 h prior, or treated with compound immediately before addition of the uptake marker.

Cells were incubated with LNP containing Cy5-labeled mRNA at a final mRNA concentration of 1 μg mL^−1^ (50 ng per well) in growth media for 2 h at 37 °C, with Hoechst 33342 in the final 10 min of incubation time. LNP and media were then removed, cells were washed twice in room temperature PBS, returned to fresh room temperature growth media and then immediately imaged live at room temperature on a Yokogawa CV8000 automated confocal microscope, using a 60× water-immersion objective (numerical aperture 1.2) at three z-planes 1 μm apart and saved as a maximum projection.

To measure transferrin receptor trafficking, cells were incubated with 8.3 μg mL^−1^ Alexa Fluor 647-transferrin (Invitrogen cat. T23366) in growth media for 10 min at 37 °C and then fixed with 4% PFA. To measure inter-nalization of 70 kDa dextran, cells were incubated with 50 μg mL^−1^ 70 kDa dextran labeled with Oregon Green (supplier, cat. D7173) in growth media for 2 h at 37 °C, washed twice with PBS and then fixed with 4% PFA and stained with Hoechst 33342 to label nuclei. Fixed samples were imaged on a Yokogawa CV8000 automated confocal microscope, using a 60× water-immersion objective (numerical aperture 1.2) at three z-planes 1 μm apart and saved as a maximum projection.

### Protein Synthesis Assay

The extent of protein synthesis was measured using the Click-iT HPG Alexa Fluor 488 Protein Synthesis Assay Kit (Thermo Fisher, cat. C10428) according to the manufacturer’s instructions. Cells were transfected with siRNA or pre–incubated with compounds for 2 h before and throughout the 30 min incubation with the HPG reagent, followed by fixation and fluorescent labeling. Samples were imaged on a Yokogawa CV8000 automated confocal microscope, using a 40× water-immersion objective (numerical aperture 1.0) at a single z-plane.

### Confocal Image Analysis and Data Processing

Images (either a single z-plane or as a maximum projection, as indicated above) were analyzed in Columbus (version 2.8.2 and 2.8.3, Perkin Elmer), data processed in GeneData Screener (version 15.0.6), Spotfire (version 7.9.2, TIBCO), and Prism (version 8, GraphPad).

For all cell imaging experiments, cell nuclei and cytoplasm were identified, and parameters measured within the total cell area, or a region within the cell area (nucleus, cytoplasm, or concentric zones radiating from the nucleus to the plasma membrane).

850 parameters pertaining to cell shape, fluorescence intensity and texture, fluorescent puncta characterization, and the spacing/clustering of cells were measured for multivariate analysis (see Methods section of the Supporting Information for analysis scripts and annotated example images). The numerical results from high-throughput image analysis of the endocytosis assays were normalized using the robust Z-score to the assay neutral control (RISC-free for siRNA, or DMSO for compounds) using Genedata Screener software. This normalization method compares each assay technical replicate to the median and variation of the technical replicates treated with the neutral control. For siRNA treatments, the neutral control was the RISC-free siRNA control, and for compounds this was DMSO.

### Machine Learning

Data were aggregated for each technical replicate identifier. For sample features with no measurements after aggregation, these features had their values set to 0. Although this might not have been ideal, the feature values were Z-score normalized against a neutral control. This resulted in a total of 490 samples with 850 features. Data were split into a stratified train/test split of 80/20%. The data classes were imbalanced, even after stratification, so Synthetic Minority Over-sampling Technique^[46]^ was used in an attempt to correct the imbalance. This was only applied to the training set using the python package Imbalanced-learn v. 0.6.2.^[47]^

Multiple models (Logistic regression, Random Forest, Gradient Boosted Tree, Support Vector Machines, Gaussian Process, K-Nearest Neighbor, Ridge Classifier, and Naive Bayes classifier) were initially trained on all 850 features using the python package Scikit-Learn v. 0.22.1.^[48]^ This yielded varying degrees of accuracies as measured by F1 score on the test set. Typically tree-based models yielded the best overall accuracy across both classes. When exploring models that exported feature importances or feature coefficients, it was noticed that the tree-based models were utilizing 20–50 of the 850 features. As it was dealing with high-dimensional data, 850 features for 490 samples, it was attempted to make easier for the models by removing features that were not predictive of functional delivery. This was performed on the training set using recursive feature elimination, *n*_features = 10 and a gradient boosted classifier as the estimator.

After recursive feature elimination, models were retrained and validated using the same train/test split, and the Random Forest, Gradient Boosted Tree, and K-Nearest Neighbor models were interrogated.

### Functional Delivery Assay Using Automated Widefield Microscopy

NCI-H358 cells were seeded and grown for 24 h in 384-well plates and then dosed with LNP by Echo acoustic dispenser and an additional 20 μL of complete media was added (giving a total of 50 μL per well). The mCherry expression and phase contrast images were acquired using an Incucyte S3 (Essen Bioscience) widefield microscope with 10× objective every 4 h for a total of 48 h. Image analysis was performed using the integrated Incucyte S3 2019A software: fluorescence threshold level was adjusted to a value above the background level (nontransfected cells) in order to identify mCherry-expressing cells. mCherry total integrated intensity and mean cell confluence were determined using segmentation masks applied over the fluorescence and phase contrast images, respectively.

### Statistical Analysis

All measurements were taken from separate wells on 384-well plates. Statistical analysis was performed in Prism (version 8, GraphPad). Tests are described in the corresponding figure caption.

### Cellular ATP Assay

Cell viability after siRNA transfection or 24 h compound incubation was measured with the CellTitre Glo 2.0 cell viability assay (Promega, cat. G9241), scaling the manufacturer’s protocol to 384-well plates and measuring luminescence with an Envision plate reader (Perkin Elmer). Results are shown in Figure S6 of the Supporting Information.

### RT-qPCR

Target knockdown was validated for a subset of siRNA using TaqMan RT-qPCR. Cells were reverse-transfected with siRNA as above and lysed at 72 h using the Real Time Ready Cell Lysis Kit (Roche, cat. 05943523001). RT-qPCR was performed using TaqMan primers (listed in Figure S7A, Supporting Information) and the Real-Time Ready RNA Virus Master (Roche, cat. 05619416001), using the LightCycler 480 (Roche) for detection. Results are shown in Figure S7B of the Supporting Information.

### mRNA Functional Delivery In Vivo

All in vivo procedures were conducted under the authority of a UK Project license which had been reviewed and approved by an Animal Welfare and Ethical Review Body in compliance with EU Directive 2010/63/EU before any work was carried out. Wild-type female BALB/c mice (6–8 weeks of age) were purchased from Charles River, UK and housed at the AstraZeneca animal facility (Babraham Research Campus). All work was carried out to Home Office U.K. ethical and husbandry standards, under the authority of an appropriate project licence. Mice were randomized and grouped for treatment. LNP formulations encapsulating luciferase mRNA were administered by intravenous injection at a dose of 0.25 mg kg^−1^. Six hours post injection, mice were administered 0.1 mL (15 mg mL^−1^) XenoLight d-Luciferin (Cat. 122799, Perkin Elmer) intraperitoneally, anaesthetized and imaged as average radiancy (p/s/cm^2^/sr) in an IVIS spectrum imager (Perkin Elmer) (15 min after administration of d-Luciferin). Statistical analysis was carried out using GraphPad Prism v.8.1.1. Difference in group mean radiance was assessed by one-way ANOVA followed by a Tukey’s multiple comparison test (significant *p*-value < 0.05).

## Supplementary Material

Supp Mat

## Data Availability

The data that support the findings of this study are available in the supplementary material of this article.
