# Supplementary material for: Understanding Intracellular Biology to Improve mRNA Delivery by Lipid Nanoparticles
Source: Small Methods. Author manuscript; Available in PMC 2023 Oct 6. (PMC7615154; doi:10.1002/smtd.202201695)
Supplement: Supp Mat [file EMS188574-supplement-Supp_Mat.pdf]

small methods

Supporting Information

for *Small Methods*, DOI 10.1002/smtd.202201695

Understanding Intracellular Biology to Improve mRNA Delivery by Lipid Nanoparticles

Morag Rose Hunter, Lili Cui, Benjamin Thomas Porebski, Sara Pereira, Silvia Sonzini, Uchechukwu Odunze, Preeti Iyer, Ola Engkvist, Rebecca Louise Lloyd, Samantha Peel, Alan Sabirsh, Douglas Ross-Thriepland, Arwyn Tomos Jones and Arpan Shailesh Desai*

SUPPLEMENTARY FIGURES

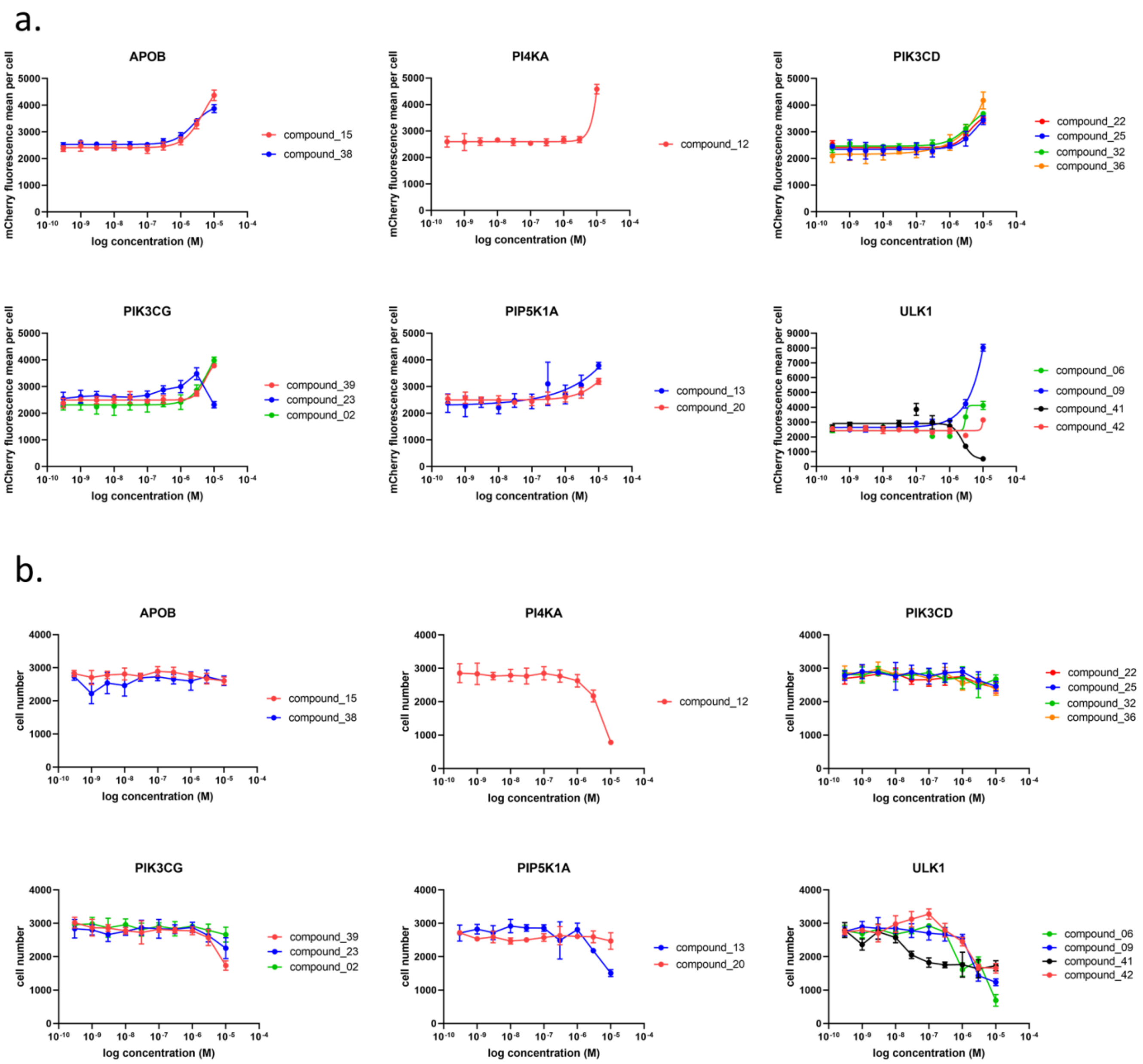

Supplementary figure 1: Compounds identified during screening as increasing functional delivery of mRNA. NCI-H358 cells were co-dosed with compound and mRNA-LNP, and incubated for 24 hours before confocal imaging (9 sites per well, 20x magnification). Compounds are plotted according to their annotated protein target. (a.) Functional delivery performance, as measured by mean mCherry fluorescence per cell; (b.) Cell number (total cells imaged per well). Mean and standard deviation, n=4.

a.

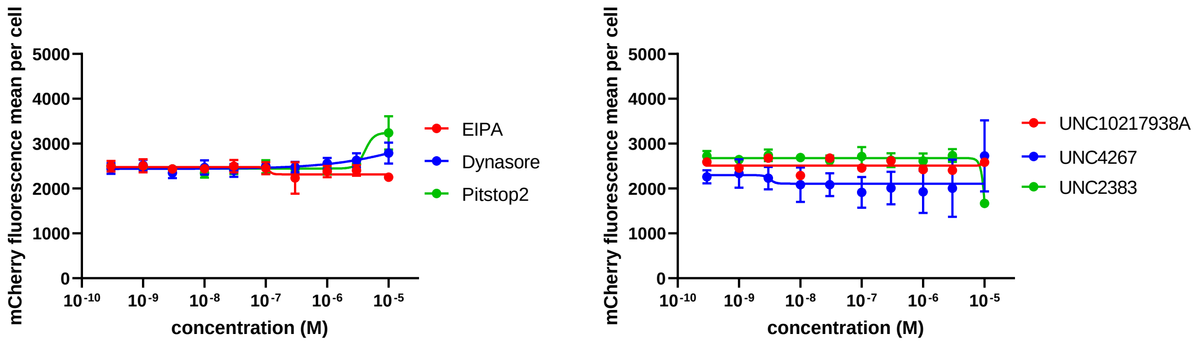

b.

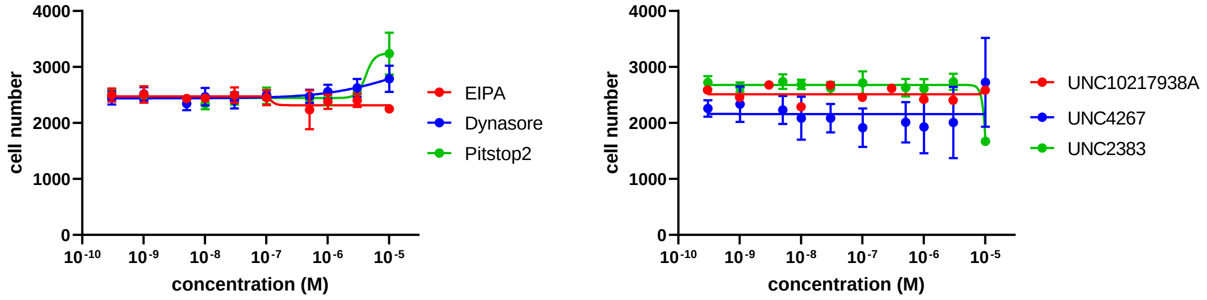

Supplementary figure 2: Previously published compounds included in compound screen. NCI-H358 cells were co-dosed with compound and mRNA-LNP, and incubated for 24 hours before confocal imaging (9 sites per well, 20x magnification). (a.) Functional delivery performance, as measured by mean mCherry fluorescence per cell; (b.) Cell number (total cells imaged per well). Mean and standard deviation, n=4.

a.

	Model	
Accuracy for each functional delivery class	Random forest	Gradient boosted tree
Neutral	0.85	0.83
Positive	0.65	0.67
Overall	0.79	0.78

b.

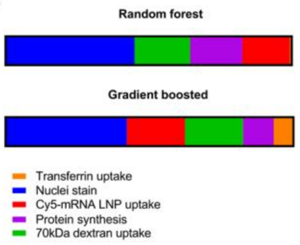

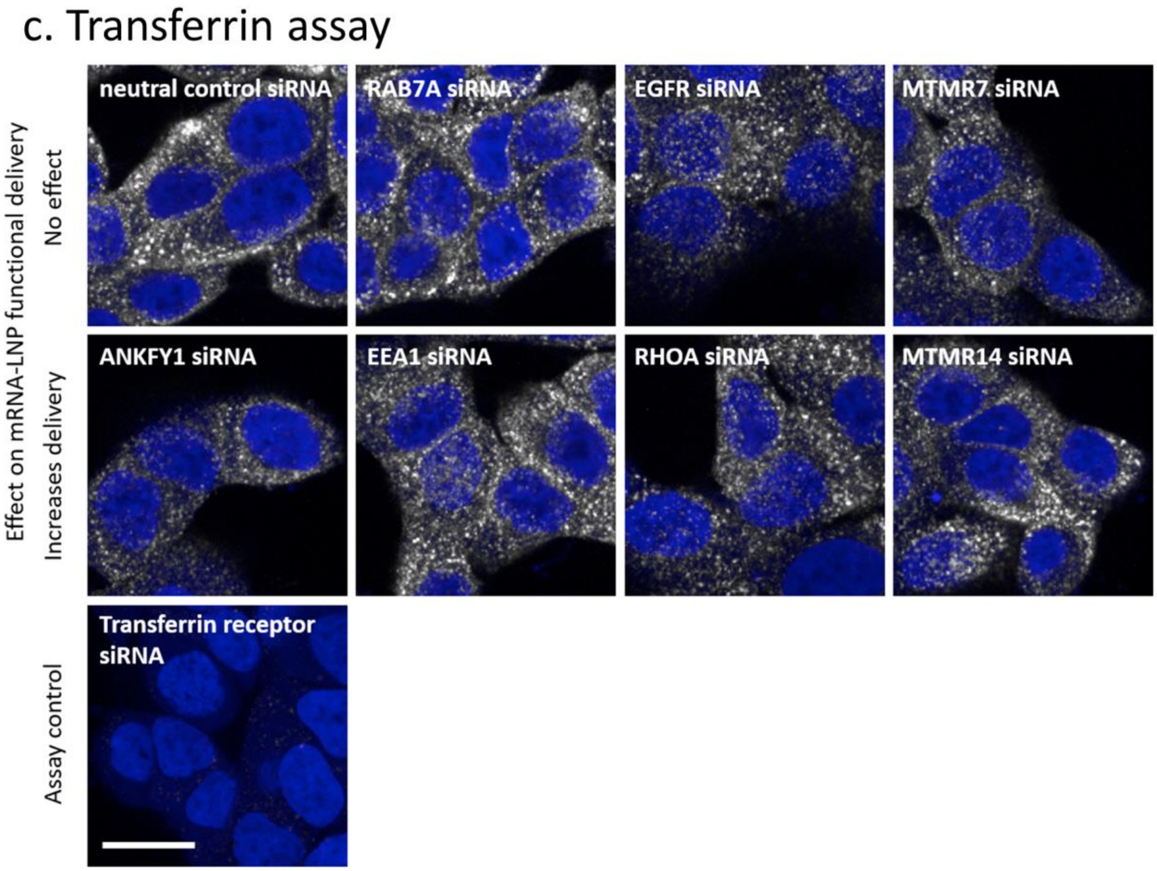

Supplementary figure 3: Further information: Machine learning models utilising all 850 features. (a-b.) Machine learning models were made utilising all 850 cell phenotypic features. (a.) Performance of machine learning models, (b.) The distribution of the top 100 weighted features of random forest and gradient boosted tree models described in A. (c.) Uptake of transferrin (white) in selected siRNA-treated cells (nuclei, blue). Scale bar 20 μm.

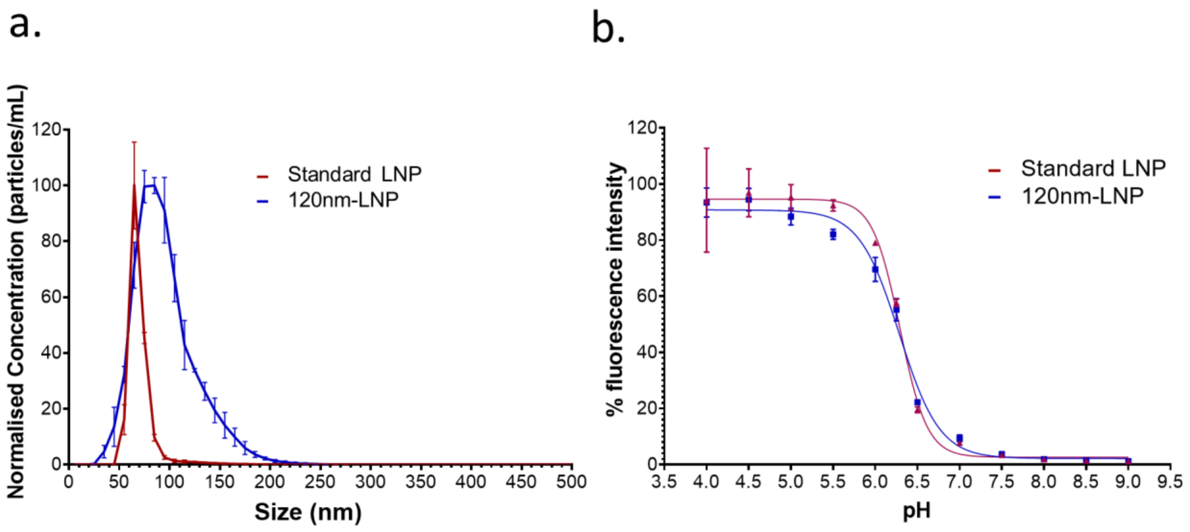

c.

LNPs		LNP composition (molar%)				Characterisation	
Size category	DSPC/area	MC3	DSPC	Chol	DMG-PEG2k	Z-ave (d.nm)	PDI
Standard	0.4	50	6.5	42	1.5	64.6	0.107
	0.5	50	8.5	40	1.5	65.9	0.098
	0.6	50	10	38.5	1.5	66.2	0.062
	0.9	50	12	36.5	1.5	78.1	0.039
	1.3	50	22	26.5	1.5	73.7	0.175
120nm-LNP	0.7	50	6.5	42	1.5	120.1	0.163
	1.0	50	8.5	40	1.5	120.6	0.163
	1.0	50	10	38.5	1.5	120.5	0.120
	1.1	50	12	36.5	1.5	120.1	0.166
	1.5	50	14.5	34	1.5	122.1	0.155
	3.0	50	22	26.5	1.5	152.7	0.171

Supplementary figure 4: Characterisation of LNP formulations. (a.) Size distribution by nanoparticle tracking analysis (NTA); (b.) pKa measurement of the LNP; (c.) Composition and characterisation of LNP when modifying both size and surface composition.

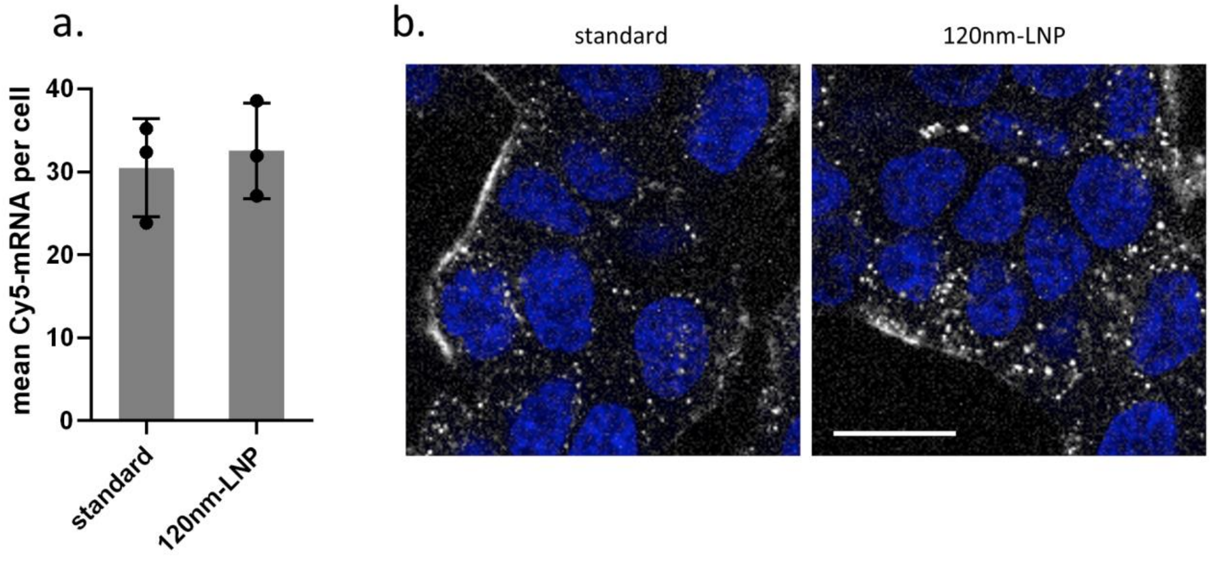

Supplementary figure 5: Uptake of standard and 120nm-LNP in H358 cells. NCI-H358 cells were incubated with Cy5-mRNA formulated into either the standard or 120nm-LNP for 2 hours. (a.) Quantification of Cy5-mCherry fluorescence per cell, mean + standard deviation, n=3; (b.) Representative images showing nuclei stained with Hoechst (blue) and Cy5-mRNA (white). Scale bar 20 μm.

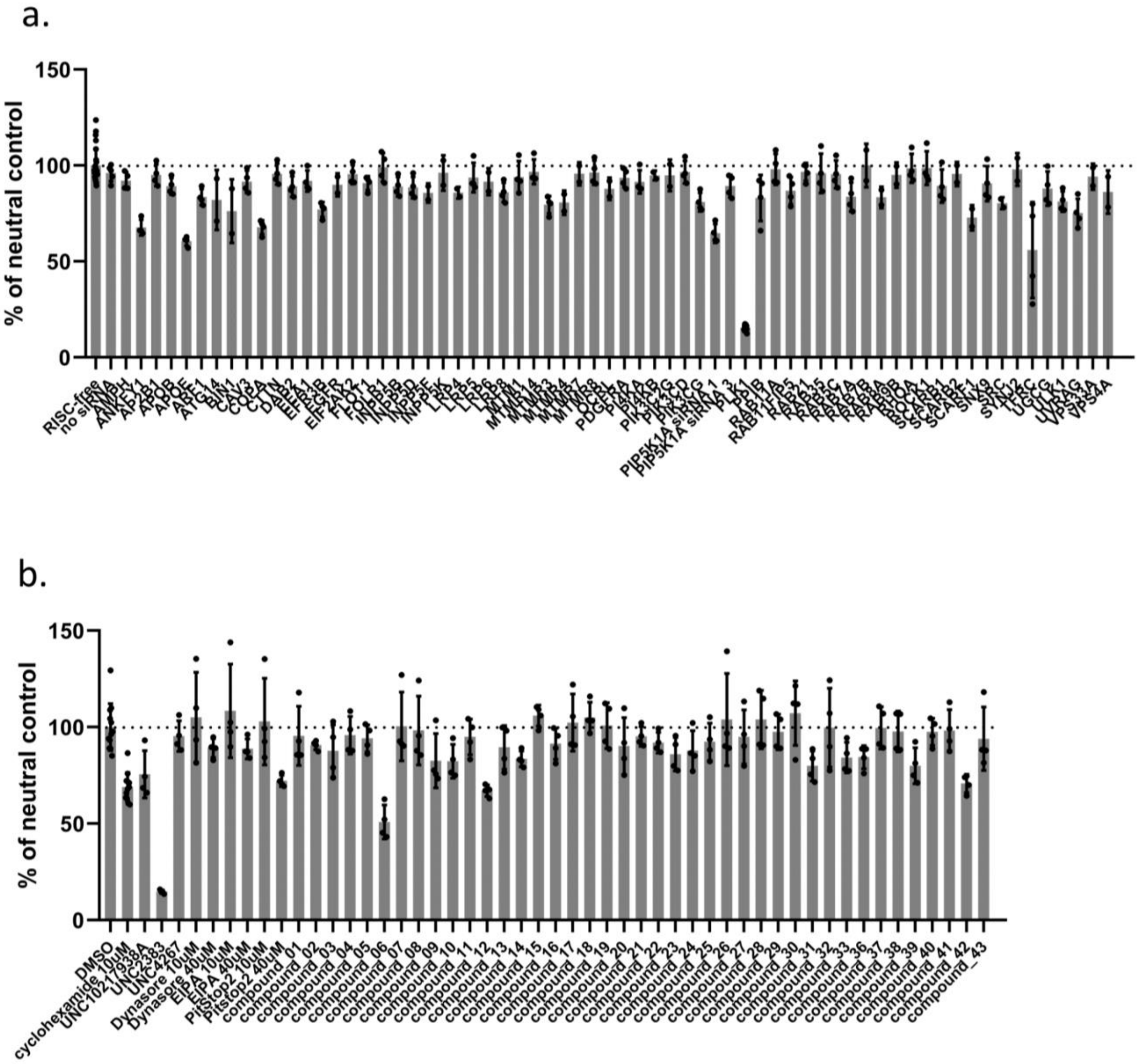

Supplementary figure 6: Toxicity of siRNA and compounds. Toxicity, as measured by cellular ATP concentrations, normalised to neutral control (100%), for cells transfected with siRNA for 72 hours (a.), or treated with compound for 24 hours (b.). Mean + standard deviation, n=2-25.

a.

Target	Taqman assay ID
ANKFY1	Hs01019630_m1
APOB	Hs00181142_m1
APOE	Hs00171168_m1
DAB2	Hs01120074_m1
EEA1	Hs00929215_m1
FLOT1	Hs00195134_m1
PI4KA	Hs01021095_m1
PIK3CD	Hs00192399_m1
PIP5K1A	Hs05027626_m1
RAB7A	Hs01115139_m1
PPIB (endogenous control)	Hs00168719_m1

b.

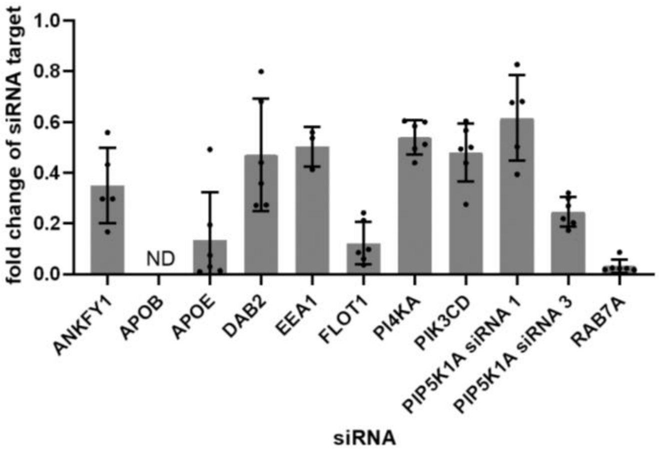

Supplementary figure 7: Knockdown efficiency of siRNA. The knockdown efficiency of a selection of siRNA conditions in NCI-H358 cells was assessed by RT-qPCR. (a.) Taqman primer/probe sets used. (b.) Fold change in mRNA concentration, relative to cells transfected with the RISC-free neutral control siRNA (which has a value of 1). Mean + standard deviation, n=3-6. ND = not detected in RISC-free or siRNA-transfected cells.

Supplementary table 1: Annotated targets of compounds identified for testing in this study.

Targets	Total compounds	Total selected
PIK3CD	3721	30
PIK3CG	1385	30
ULK1	299	26
PIP5K1A	260	26
ROCK1	179	26
PI4KA	153	26
PDGFRA	117	26
FOLR1	6	6
APOB	3	3
LRP5	1	1
